# Discovery of Azurin-Like Anticancer Bacteriocins from Human Gut Microbiome through Homology Modeling and Molecular Docking against the Tumor Suppressor p53

**DOI:** 10.1155/2016/8490482

**Published:** 2016-04-30

**Authors:** Chuong Nguyen, Van Duy Nguyen

**Affiliations:** ^1^Theoretical Physics Research Group, Ton Duc Thang University, Ho Chi Minh City, Vietnam; ^2^Faculty of Applied Sciences, Ton Duc Thang University, Ho Chi Minh City, Vietnam; ^3^Institute of Biotechnology and Environment, Nha Trang University, Nha Trang, Khanh Hoa, Vietnam

## Abstract

Azurin from* Pseudomonas aeruginosa* is known anticancer bacteriocin, which can specifically penetrate human cancer cells and induce apoptosis. We hypothesized that pathogenic and commensal bacteria with long term residence in human body can produce azurin-like bacteriocins as a weapon against the invasion of cancers. In our previous work, putative bacteriocins have been screened from complete genomes of 66 dominant bacteria species in human gut microbiota and subsequently characterized by subjecting them as functional annotation algorithms with azurin as control. We have qualitatively predicted 14 putative bacteriocins that possessed functional properties very similar to those of azurin. In this work, we perform a number of quantitative and structure-based analyses including hydrophobic percentage calculation, structural modeling, and molecular docking study of bacteriocins of interest against protein p53, a cancer target. Finally, we have identified 8 putative bacteriocins that bind p53 in a same manner as p28-azurin and azurin, in which 3 peptides (p1seq16, p2seq20, and p3seq24) shared with our previous study and 5 novel ones (p1seq09, p2seq05, p2seq08, p3seq02, and p3seq17) discovered in the first time. These bacteriocins are suggested for further* in vitro* tests in different neoplastic line cells.

## 1. Introduction

As one of the most deadly diseases worldwide, cancer is involved in disregulation of mammalian cell differentiation and growth. There is now no conceivable way that current drugs can prevent cancer relapse once the cancer is in remission. The common treatment of cancer is undertaking surgical resection of the tumors followed by radiation and chemotherapy [1]. There are two types of drugs that are normally used in chemotherapy, including small molecule drugs (e.g., tyrosine kinase inhibitors) and human or humanized proteins (e.g., monoclonal antibodies). However, these “one drug-one target” therapies can cause the most devastating side effects on the growth of normal cells and lead to the rapid resistance to drugs developed by the cancer cells using alternate pathways for growth or using efflux pumps to pump out drugs [2]. Therefore, new therapies for cancer drug discovery using multitargeted approaches to overcome resistance, toxicity, and side effects are urgently needed.

Over the past centuries, a phenomenon of spontaneous regression of tumors associated with bacterial infections has been observed [3]. One of the most well-known treatments based on this phenomenon was reported in late 1890s by an American physician, Coley [4]. He observed the relationship between bacterial infection and cancer regression, which led to the discovery of a killed bacterial vaccine for cancer, known as “Coley's toxin” [3]. This suggested renewed interest in the development of new therapeutic anticancer modalities based on the use of live bacteria and their purified products including bacterial toxins, proteins, peptides, and enzymes. Recently, a number of bacterial proteins and peptides have been described to exert an anticancer activity at preclinical level toward diverse types of cancer cells [1]. Among them, bacteriocins are antimicrobial peptides or proteins ribosomally synthesized by bacteria to inhibit the growth of the similarly or closely related bacterial strains (narrow spectrum) and sometimes against a wide spectrum of species. They have been looking for a positive health benefit to the host including human, livestock, aquaculture animals, and some plants [5]. Bacteriocins promise to be effective therapeutic agent and their biochemical properties have been studied; their antineoplastic capability has also identified after its discovery in the late 1970s by using crude bacteriocin preparation toxic to mammalian cells [6]. Common bacteriocins like pyocin, colicin, pediocin, and microcin have been shown to possess inhibitory properties against different neoplastic line cells [5].

Among well-known protein anticancer agents in bacteria, there are immunotoxins and several bacterial proteins including* Mycobacterium bovis* MPT63, arginine deiminase from* Mycoplasma arginini*, lipidated azurin (laz) from* Neisseria meningitides*, and azurin from* Pseudomonas aeruginosa *[1]. The latest one, azurin, is an important bacteriocin, a member of the cupredoxin family of redox proteins, which becomes a potential anticancer drug because of some of its unique properties. Azurin can preferentially penetrate human cancer cells and exerts cytostatic and apoptotic effects with no apparent activity on normal cells [7, 8]. Azurin can directly interact and stabilize the tumor suppressor p53 [7]. The azurin domain responsible for its specific entry in cancer cells was demonstrated that it spans residues 50–77 (termed p28) and adopts an amphipathic alpha helical conformation [9]. Cell penetration is not accompanied by membrane disruption, which could cause cell death. Preclinical evaluation of pharmacokinetics, metabolism, and toxicity of azurin-p28 was evaluated [10], establishing it as nonimmunogenic and nontoxic in mice and nonhuman primates. Moreover, the protein-protein interactions between azurin and p53 have recently been analyzed by bioinformatics and atomic force microscopy [11–13].

Interestingly, not only does azurin have anticancer activity but also it strongly inhibits host cell invasion by the AIDS virus HIV-1 [14], the malarial parasite* Plasmodium falciparum* [14], and the toxoplasmosis-causing parasite* Toxoplasma gondii* [15]. Thus azurin is believed to be a weapon used by* P. aeruginosa* to keep invaders of the human body for long term residence without harming or exerting any toxicity to the host [1]. This also suggests that azurin may be specific for tumors in the organs where* P. aeruginosa* normally resides during infection. In fact,* Neisseria meningitides* produces an azurin-like protein called laz (lipidated azurin) with a 127 amino acid moiety with 56% amino acid sequence identity to* P. aeruginosa* azurin. Several US patents have been issued to cover the use of azurin and laz in cancer therapies [16], and azurin has shown significant activity, as well as enhancement of the activity of other drugs, in oral squamous carcinoma cells [17].

The very important question is whether azurin is the only bacteriocin produced by* P. aeruginosa* as an anticancer weapon or whether there are other bacteriocins, produced by other bacteria with the ability to cause chronic infections and have long term residence in human bodies, as well as defending the body from invaders such as viruses and parasites. It is, thus, interesting to note that azurin is not the only anticancer bacteriocins produced by human microflora. In fact, their antineoplastic properties have been inadequately revealed in the late 1970s by using crude bacteriocin preparation toxic to mammalian cells. Nowadays, purified bacteriocins are available and have shown inhibitory properties toward diverse neoplastic line cells. Pyocin, colicin, pediocin, and microcin are among bacteriocins reported to present such activity [5, 18].

Although bacteriocins have been found in many major lineages of bacteria and some members of the Archaea, more and more new bacteriocins with new characteristics and origins are still awaiting discovery. By now, bacteriocins have mainly been derived from the lactic acid bacteria with mostly fermented food origins. Besides, colicins from* E. coli* were used as model Gram-negative bacteriocins. There were only a few basic researches on noncolicin bacteriocins of human origins and bacteriocins with killing activity against eukaryotic and human cells [5].

Here, we hypothesize thatbacteria from human microflora, especially pathogenic and commensal bacteria, with long term residence in human body can produce azurin-like bacteriocins as a weapon to protect their habitat from cancers. In our previous work [19], putative bacteriocins have been screened from complete genomes of 66 dominant bacteria species in human gut microbiome and subsequently characterized by subjecting them as functional annotation algorithms with azurin as control. We have predicted a number of bacteriocins possessed functional properties very similar to those of azurin [19]. However, the study was limited to qualitative assessment of the bacteriocins at sequence-level only. In addition, the hydrophobicity of the peptides, which is suggested to play an important role on anticancer activity, has not been addressed. Therefore, in this study, we performed a system of quantitative analyses including functional prediction (using a scoring function to evaluate) and hydrophobic percentage calculation to identify azurin-like bacteriocins. Next, to extend our analysis at structural level, we performed structural modeling and molecular docking study of bacteriocins of interest against protein p53, a cancer target. These analyses provided us more reliable data to identify azurin-like bacteriocins with potential anticancer activity.

## 2. Materials and Methods

### 2.1. Screening of Potential Bacteriocin Sequences from Human Gut Microbiome

**Figure 1:**
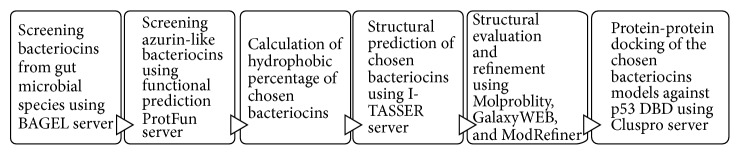
Flowchart illustrating the process of identification and subsequent characterization of hypothetical anticancer bacteriocins from gut microbial species.

In our previous work, hypothetical bacteriocins which possess properties similar to azurin have been suggested [19]. In summary, the complete genomes of 66 dominant species among 101 prevalent gut microbial species [20] were retrieved from the National Center for Biotechnology Information (NCBI) Databases (http://ncbi.nlm.nih.gov/). They were then scanned using the BAGEL web server (http://bagel.molgenrug.nl/) [21] in order to identify putative genes encoding bacteriocins. The output of BAGEL is the protein sequences of hypothetical bacteriocins. The sequences were saved in FASTA format for subsequent analysis (Figure 1).

### 2.2. Screening Azurin-Like Bacteriocins Using Functional Prediction ProtFun Server

The hypothetical bacteriocins sequences were subjected to the web server ProtFun 2.2 (http://www.cbs.dtu.dk/services/ProtFun-2.2/) [22, 23]. The ProtFun 2.2 server produces* ab initio* predictions of protein function from sequence. The* ab initio* feature is important for hypothetical bacteriocins because most of the sequences predicted from BAGEL server in previous step are not yet available in any sequence database. For each input sequence, the server predicted cellular function (F), enzyme class (EZ), and gene ontology (GO) category. The scores of the prediction consist of two numbers: the probability and the odds number. The first number is the estimated probability that the entry belongs to the class in question, being influenced by the prior probability of that class. The second number represents the odds that the given sequence belongs to that class/category. It is independent of the prior probability.

In this work, we use the odds number to rank our hypothetical bacteriocins using azurin as a control. To estimate the similarity between each hypothetical bacteriocin sequence and azurin, we collect the predicted odds number that the sequence belongs to the same category with azurin. The score of similarity of a sequence with azurin is estimated using logarithm of production of three odds numbers:

(1) Scoreseq=log⁡oddsseq ∣ azurin_F×oddsseq ∣ azurin_EZ×oddsseq ∣ azurin_GO.

The higher the score, the higher the odds that the sequence belongs to the same categories with the azurin. All the sequences with the score higher than 0 were selected for further analysis.

### 2.3. Calculation of Hydrophobic Percentage

Hydrophobic interaction is relatively stronger than other weak intermolecular forces such as Van der Waals interactions or hydrogen bonds and is suggested to play an important role for membrane permeabilization and in antitumor activity [24]. Therefore, information about hydrophobic amino acids in short peptides is considered to be among the key criteria for ranking.

The sequences from previous step were subjected to calculations of hydrophobic percentage. In other words, these calculations give us the percentage of hydrophobic amino acids with respect to the total amino acids of the sequence. The residues considered as hydrophobic are Phenylalanine (Phe, F), Isoleucine (Ile, I), Leucine (Leu, L), Methionine (Met, M), Valine (Val, V), Tryptophan (Try, W), Glycine (Gly, G), Cystein (Cys, C), and Alanine (Ala, A). All hypothetical bacteriocins as well as azurin and p28-azurin were subjected to hydrophobic percentage calculation. Those bacteriocins which have hydrophobic percentage similar to azurin are considered to be promising azurin-like anticancer bacteriocins and selected for next step.

### 2.4. Protein Structure Prediction

Because 3D structures of all the bacteriocins selected from previous step have not been available in RCSB Protein Data Bank yet; modeling of these peptides was done by using I-TASSER server (http://zhanglab.ccmb.med.umich.edu/I-TASSER). I-TASSER is a hierarchical bioinformatics method for predicting three-dimensional structure of protein molecules from amino acid sequences [25]. This method detects template structures from the Protein Data Bank (PDB) by multiple threading approaches. The full-length atomic models are constructed by iterative template fragment assembly simulations using replica exchange Monte Carlo method. I-TASSER (as previously called “Zhang-Server”) is ranked as the best server for protein structure prediction in recent Critical Assessment of Structure Prediction (CASP) experiments [25].

All chosen sequences (in FASTA format) were submitted to I-TASSER server. For each sequence, the server predicted a number of models and ranked them by *C*-score. The *C*-score is typically in the range of (−5, 2), where a *C*-score of higher value signifies a model with a high confidence. The model with the highest *C*-score was selected and refined by GalaxyWEB [26] and ModRefiner [27] servers. The models before and after refinement were all validated for their backbone conformation geometry and the residue contact using MolProbity [28].

### 2.5. Docking Potential Anticancer Bacteriocins against Cancer Target p53

Protein-protein docking approach was used to predict binding poses of the potential anticancer bacteriocins selected from the previous step to the common cancer target p53 DNA binding domain. The anticancer p28 peptide fragment of azurin was also docked against p53 to validate the docking method. The protein p53 core domain mutant (PDB ID 2J1X) obtained from Protein Data Bank [29] was chosen as the receptor for docking. Cluspro 2.0 [30], a fully automated web-based program for the computational docking of protein structures, was employed. Cluspro 2.0 protein-protein docking algorithm works in three main steps. In first step, it runs PIPER, based on a Fast Fourier Transform (FFT) docking method. Secondly, it used a clustering approach for the identification of near native conformations and discards the unstable clusters. Finally, a short Monte Carlo simulation was applied to judge the stability of these clusters and further refined [30]. For each peptide, the best model based on cluster size was chosen for further analysis.

## 3. Results and Discussion

### 3.1. Screening Azurin-Like Bacteriocins Using Functional Prediction ProtFun Server

Prediction of functions of azurin from* Pseudomonas aeruginosa* (uniprot ID: P00282) and all 81 putative bacteriocins using ProtFun server is calculated and shown in Table S1 (Supplementary Material available online at http://dx.doi.org/10.1155/2016/8490482). Azurin is predicted as cellular envelope (odds 9.71), nonenzyme (odds 1.147), and gene oncology of immune response (odds 5.877). In this work, the three functional categories are considered equally important. Thus, the score of similarity in (1) is the result of production of three odd numbers without any weighted coefficient for each odd number (odds product 65.5). This score helps us avoid biasing toward any particular property of the sequence.

The sequences of putative bacteriocins are ranked by odd score from high to low. The higher the score, the higher the odds that the sequence belongs to the same categories with the azurin. There are 28 sequences with odds score greater than 0. They are considered most similar to azurin among all 81 putative bacteriocins. Considering each odd score, p3seq02 has largest immune response odds at 5.0, which is smaller than that of azurin. However, p1seq04 has largest nonenzyme odds (1.2) among others, while p2seq07 has the largest cellular envelope odds (10.2) being the top hit of the odds product at 25.5. Although p3seq02 has the largest immune response odds compared to the p2seq07 (4.1), it has much lower nonenzyme odds and cellular envelope odds compared to p2seq07 and azurin.

The probability score for each sequence was also calculated. We found that there is a strong correlation between the odds score and probability score with correlation coefficient *R* = 0.998 (Figure S1, Supplementary Material). This result suggests that the probability score can also be used as odds score to rank the similarity of bacteriocins and azurin.

Thus, in this first step we identified 28 novel putative bacteriocins possessed functional properties very similar to that of azurin. ProtFun results have predicted that azurin has cell envelope function while our bacteriocins expressed the same properties, revealing a great chance of possessing cancer cell attack activity similar to that of azurin. The more important functional characterization, which was to predict the propensity of the bacteriocins, was enzymatic activity. The low propensity of azurin to have enzymatic activity indicates that it has little chance of interacting with any other substrates or altering the normal cellular kinetics. This is consistent with the special feature of azurin which targets human cancer cells without exerting activity on normal cells [7–9]. Like azurin, our bacteriocins showed immune responses which have very low chances of drug-induced adverse reactions of type B which comprise idiosyncratic and immune-mediated side effects [31]. These results signify that our selected bacteriocins have great chance of having anticancer activity similar to that of azurin.

### 3.2. Calculation of Hydrophobic Percentage

It has been suggested that the hydrophobicity of a peptide plays a crucial role in the mechanism of action against cancer cells, which should be taken into account in the design of potential anticancer peptides [32, 33]. The anticancer activity of a peptide has been shown to be correlated with the peptide hydrophobicity which means that increasing hydrophobicity leads to the increase of anticancer activity [33]. Peptides with higher hydrophobicity are suggested to enter deeper into the hydrophobic core of the cell membrane, causing stronger activity of disrupting the cancer cell membrane [33]. On the other hand, the peptide specificity against cancer cells and normal cells depends on the hydrophobicity in different manner. Too low or too high hydrophobicity reduces the specificity of the peptide against cancer cells [33]. Thus, a designed peptide should have the hydrophobicity in the range that has the capability of entering cells as well as the specificity against cancer cells.

One common index that can be used to measure the hydrophobicity is the percentage of hydrophobic residues in each sequence. The hydrophobic percent of azurin sequence and p28-azurin (amino acids 50 to 77 of azurin) is 49% and 46%, respectively. Azurin and p28-azurin have been shown to have entry specificity in cancer cells and prevent cancer cell growth by interfering in multiple pathways by which cancer cells grow [34]. Interestingly, p18-azurin (amino acids 50 to 67 of azurin) has been shown to be responsible for the entry of azurin into human cancer cells but not for the inhibition of cancer cell proliferation [35]. The hydrophobic percent of p18-azurin is 61% that is much higher than the hydrophobic percents of azurin and p28-azurin. Thus, to screen out putative bacteriocins with anticancer potential as azurin, the sequences that have hydrophobic percentage from 44% to 51% are selected. This corresponds to the fact that hydrophobic percentage of chosen bacteriocins is within 2% deviation from that of azurin or p28-azurin.

Among 28 putative azurin-like bacteriocins, there are 14 sequences that satisfy these criteria (Table 1). In which, 10 of them are small peptides (7 Sactipeptides, 2 Lasso peptides, and 1 UviB class II) and 4 of them are large proteins (all Zoocin A type). These sequences are found in 7 bacterial species including* Anaerotruncus colihominis* (1 sequence),* Bacteroides vulgatus* (2 sequences),* Clostridium hathewayi* (reclassified as* Hungatella hathewayi*) (1 sequence),* Clostridium nexile* (6 sequences),* Dorea longicatena* (1 sequence),* Eubacterium ventriosum* (1 sequence), and* Ruminococcus *sp. (2 sequences). Only* Bacteroides vulgatus* is a species within the Order Bacteroidales, whereas all remaining species belong to the Order Clostridiales. A molecular genetic analysis of rDNA amplicons generated directly from a human faecal sample showed that more than 90% of the flora could be assigned to three major phylogenetic lineages (the* Bacteroides, Clostridium coccoides,* and* Clostridium leptum* groups) [36]. Thus our bacteriocins were produced by the most dominant bacterial species in human gut. Interestingly, most of putative azurin-like bacteriocin-producing species are considered as pathogenic bacteria, which fits well with our hypothesis. The results are also in agreement with cases of azurin-producing* Pseudomonas aeruginosa* and laz-producing* Neisseria meningitides.*

### 3.3. Structural Prediction of Chosen Bacteriocins

I-TASSER is one of the most popular online servers for automated protein structure prediction. The accuracy of generated structural models of I-TASSER is comparable to the best human-expert guide modeling [25]. This server can be used for sequences with or without similar folds in the Protein Data Bank (PDB) library [37]. Thus, tertiary structures of 14 putative bacteriocin sequences were predicted using I-TASSER server [25] and then refined by GalaxyWEB [26] and ModRefiner [27] servers. Predicted structures are shown in Figure S2 (Supplementary Material). The percentage of ramachandran favored residues and rotamer favored residues of 14 refined models is 90.2% and 97.4% on average (Table S2, Supplementary Material).

These data also showed that, for each bacteriocin, the quality of the model improves significantly after refinement. The MolProbity scores of all refined models were also calculated. MolProbity score combines the clash score, rotamer, and Ramachandran evaluations into a single score, normalized to be on the same scale as X-ray resolution [28]. The scores of all refined models are below 1.5 indicating that our models have the quality of corresponding X-ray resolution [28].

A new database of structurally annotated therapeutic peptides, SATPdb (http://crdd.osdd.net/raghava/satpdb/), which is curated from 22 public domain peptide databases/datasets, holds 19192 unique experimentally validated therapeutic peptide sequences including 1099 anticancer ones [38]. However, using Blast search on whole this database with *E*-value = 1 applied for 14 putative bacteriocins of our interest, no hits were found, with an exception of p3seq24 from* Clostridium hathewayi* DSM 13479. The p3seq24 has 37% identities (*E*-value = 0.002) with* Clostridium perfringens* UviB (Bactibase ID: BAC090), which is predicted as an antimicrobial and antibacterial bacteriocin. This indicates that all our putative bacteriocins are novel to previously experimentally validated therapeutic peptides.

### 3.4. Docking Potential Anticancer Bacteriocins against Cancer Target p53 DNA Binding Domain

Molecular docking along with atomic force microscopy studies has recently revealed the binding interface of p28 and the DNA-binding domain (DBD) of p53 [39]. The authors suggested that the L1 loop (aa 112–124), a region within the S7-S8 loop (aa 214–236) and Thr 140, Pro 142, Gln 144, Trp 146, Arg 282, and Leu 289 of the p53 DBD are potential sites for p28 binding.

**Figure 2:**
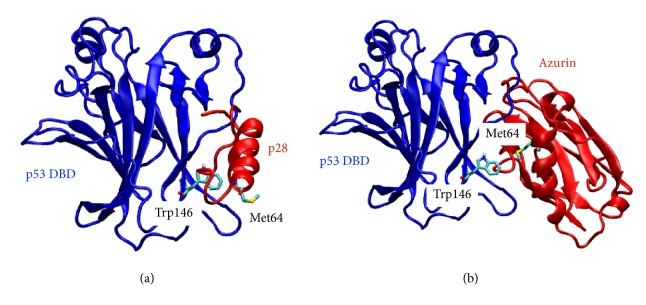
Three-dimensional configurations of docking models of p53 DBD with p28-azurin (a) and with azurin (b).

We performed molecular docking of p53 DBD (PDB code 1TUP chain B) with p28-azurin using Cluspro server [30]. Cluspro carried out a cluster analysis and the best one had more cluster members and lowest energy compared to other members. We selected the first cluster for validation and further analysis (Figure 2(a)). We have found very similar binding sites which are L1 loop (aa 104, 109–116, 123–134), an amino acid within S7-S8 loop (aa 228), aa 141–148, 268, 282, and 286 (Figure S3, Supplementary Material). Considering that we carried out docking without any prior knowledge of binding site of this complex, these results show a striking overlap between our predicted binding site and that of the authors [39].

One should note that the conformation of a peptide in solution could be different from that observed when it is embedded in the protein. Indeed, the p28-azurin reported in [39] was subjected to molecular dynamics simulation in aqueous solution before docking. The final conformation from molecular dynamics simulation is slightly different from the conformation used in this study, mainly at two terminals of the peptide. However, as mentioned above, our prediction of binding interface between p28 and p53 DBD is in good agreement with their prediction. This suggests that the configuration of p28 observed in azurin is likely stable in aqueous solution.

Nevertheless, due to the computational cost, performing molecular dynamics simulation may not be able to address all the possible conformations of a peptide. In addition, peptide configuration might be changed upon binding to the receptor. Thus one could do peptide flexible docking that searches for the binding interface of the complex while allowing for full flexibility of the peptide. However, this approach still remains a computational challenge owing to the high torsional flexibility of peptides. Recent benchmark [40] has shown that flexible docking of a peptide of up to four amino acids length can be reproduced quite accurately. However, when a peptide has more than four amino acids, the accuracy of prediction declined dramatically [40]. Another benchmark for short peptides (smaller than five) has shown that peptide rigid docking is even more successful than peptide flexible docking [41]. Because all the bacteriocins of interest in this study have more than 30 amino acids, a well-tested program that is able to predict the binding pose of protein-peptide complexes such as Cluspro [30] is therefore a suitable choice.

We also carried out molecular docking of p53 DBD with full-length azurin (PDB code 3U25 chain A) for validation of the method with the whole protein (Figure 2(b)). The binding interface includes L1 loop (aa 113–124), a region within the S7-S8 loop (aa 224–235), 140, 144, 146, 198, and 199 (Figure S4, Supplementary Material). This binding interface highly overlaps with previous studies as well as the binding interface predicted for p28. Importantly, the binding region of azurin includes the fragment of p28 (aa 56–72) in which the residue Met 64 is found to bind with p53 DBD Trp 146. Interestingly, even the binding poses of p28 alone and p28 within azurin do not exactly overlap, the residue Met 64 in both models binds to the same residue Trp 146 of p53 (Figure 2). In total, we found in our models 8 amino acids of p53 that bind with both azurin and p28-azurin. They include Phe 113, Leu 114, His 115, Ser 116, Cys 124, Gln 144, Trp146, and Asp 228. These results agree well with recent study of p28 in complex with p53 [39] in which these 8 residues are all found in the binding region.

**Figure 3:**
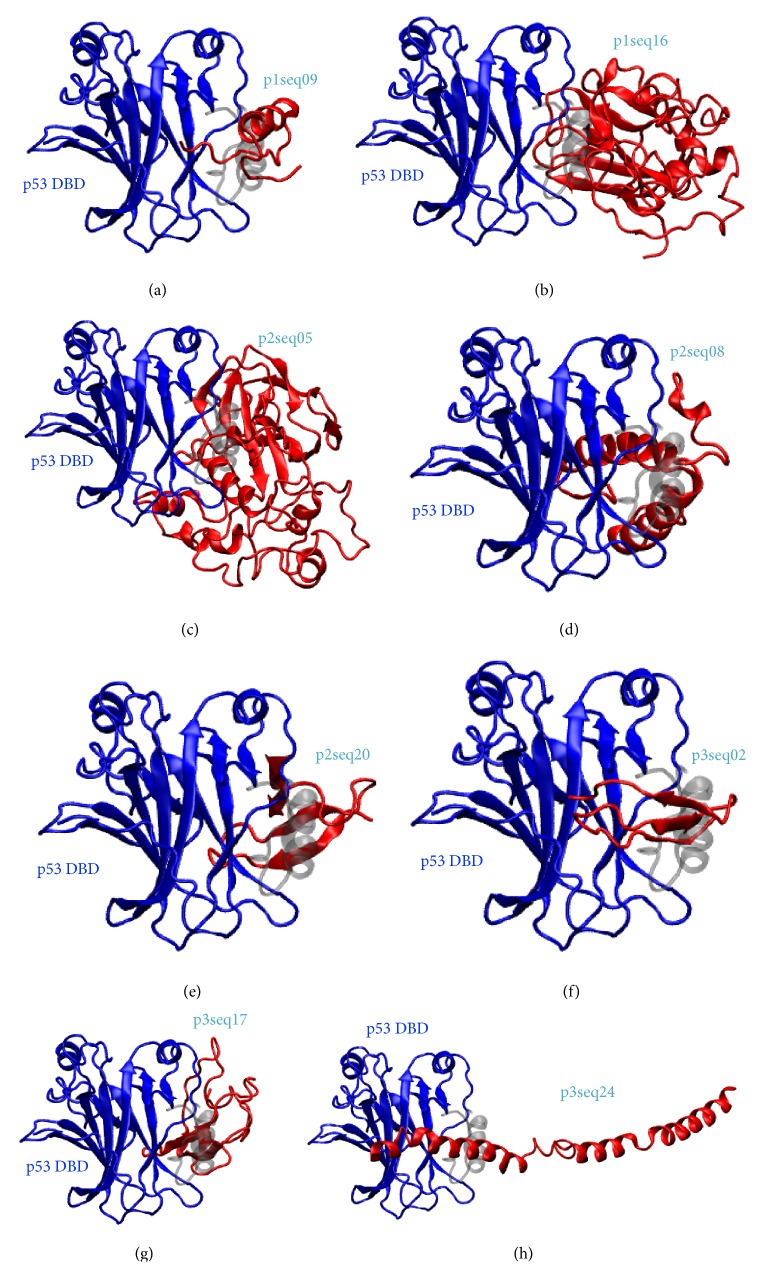
Three-dimensional docking models of 8 bacteriocins that have the highest similar binding site with p28-azurin and azurin when they bind to p53 DBD. The bacteriocins structure is shown in red, p53 DBD in blue, and p28-azurin in grey for reference.

We have performed molecular docking for all models predicted from previous step using Cluspro [30]. For each complex, we select the first cluster for further analysis. To identify the bacteriocins with anticancer property like azurin or p28-azurin, we assume that they should bind in the same region of p53 as that of azurin or p28-azurin. We found 8 bacteriocins which bind in the same region as p28 and azurin (Table 2). They include p1seq09, p1seq16, p2seq05, p2seq08, p2seq20, p3seq02, p3seq17, and p3seq24. Their binding poses are shown in Figure 3. Details of amino acid of p53 DBD involved in binding interface with each bacteriocin are listed in Table S3 (Supplementary Material).

In our previous study, we identified 14 final putative bacteriocins based on the qualitative assessment of the functional properties similar to azurin and laz (lipidated azurin produced by* Neisseria meningitides*) [19]. In the present work, 8 final candidates were identified based on a system of quantitative analysis of the azurin and p28-like functional prediction scoring with hydrophobicity added, structural modeling, and molecular docking studies against p53. Only three final bacteriocins (p1seq16, p2seq20, and p3seq24) were shared by both two approaches, and interestingly, five novel putative bacteriocins (p1seq09, p2seq05, p2seq08, p3seq02, and p3seq17) with potential anticancer activity were discovered in the present study, indicating the similar and improved results from the quantitative and structure-based analyses.

Similarly, using bioinformatics approach, the entire genome of a human commensal bacterium* Lactobacillus salivarius* was scanned for putative bacteriocins and potentially anticancer bacteriocins were screened through structure prediction and docking studies against the common cancer targets p53, Rb1, and AR with azurin as control. The results have revealed that Lsl_0510 possessed highest binding affinity toward all the three receptors [42]. In the present work, for the first time, a multi-genome-scale screening, homology modeling, and molecular docking study of putative bacteriocins from all 66 dominant bacteria species in human gut microbiome was performed to finally identify 8 candidate peptide drugs with azurin-like anticancer activity. Further* in vitro* tests were required to make them be ideal candidates for future cancer therapeutics.

In order to develop a cancer therapeutic drug, at least four important properties should be considered: (i) nontoxicity for long term use, (ii) inhibiting and killing any preformed tumor cells, (iii) preventing oncogenic transformation of normal cells to cancer cells, and (iv) being taken orally and not through intravenous injections. The three former properties are shared by p28 and probably by azurin, although azurin's toxicity and side effects in humans have not yet been assessed [1]. For the fourth feature, p28 is now given intravenously but future technological advances might overcome this problem.

In the present study, 6 of 8 final candidate bacteriocins are small peptides composed of 31 to 77 amino acids (Table 2). These peptides can be administered orally, through subcutaneous or intravenous injections, or even by inhalation. Actually, peptides that have entered the global market are composed of up to 40 or more amino acids. There are only about 11 peptides approved by FDA from 1985 to 2013 and valued more than US$ 1.0 billion in global sales, which include Copaxone, Lupron, Zoladex, Sandostatin, Lucinactant, Peginesatide, Pasireotide, Carfilzomib, Linaclotide, Teduglutide, and Lixisenatide [1]. However, only a few such as Carfilzomib are indicated for cancer therapy. Thus, along with positive results of p28-azurin in phase I clinical trials in Chicago with 15 stage IV cancer patients [43], small peptides with azurin-like anticancer activity may have promisingly opening potential in cancer therapy in the future.

The remaining two of 8 final candidate bacteriocins are larger proteins composed of 212 to 262 amino acids (Table 2). These proteins have additional domains in their structure and thus may have other cancer growth inhibitory activities that a small peptide lacks. For example, compared to p28, azurin expressed the multidomain and multivalent action to preferentially enter cancer cells and interfere in multiple steps in cancer growth, both intracellular and extracellular [1]. Azurin also have multibiological activity such as antiparasite antiviral activity, including its ability to combat AIDS [14, 15]. Even azurin has been overproduced in* Escherichia coli* Nissle 1917 probiotic cells to allow the regression of melanoma and breast tumor in the mouse model [44]. Although azurin must often be administrated through intravenous injections rather than orally, it can be chemically synthesized at a modest cost or chemically modified (e.g., insulin can be covalently linked to polyethylene glycol) to become more stable to stomach acids, easily absorbed through gut tract, stable in serum, and less immunogenic [1]. Therefore, large proteins like azurin have more domains in addition to smaller peptides like p28 that can make them a much more effective drug if their efficacy, lack of toxicity, lack of susceptibility to resistance development, and an improvement in mode of administration toward technological advances can be demonstrated in preclinical and human clinical trials.

## 4. Conclusion

Using bioinformatics approaches including functional prediction (scoring), hydrophobic percentage calculation, structural modeling, and molecular docking, at least 8 putative bacteriocins from human gut pathogenic and commensal bacteria have been found to possess functional properties very similar to those of azurin and p28-azurin with potential anticancer activities. Among them, 3 peptides (p1seq16, p2seq20, and p3seq24) have been shown from our previous study and 5 novel ones (p1seq09, p2seq05, p2seq08, p3seq02, and p3seq17) are discovered for the first time here. The results herald a new era of drug development and contribute to better human health.

If the pathogenic and commensal bacteria with long term residence in human body produce these proteins to defend their habitat from invaders such as cancers and other deadly diseases, this can lead us to identify the novel anticancer drugs from human microflora. The discovery of these drugs has just been started.

## Supplementary Material

Table S1 shows detail information of functional prediction of 81 putative bacteriocins by ProtFun server, probability scores and odd scores. Table S2 shows model assessments for the 14 most putative anticancer azurin-like bacteriocins structure predicted by I-TASSER. Table S3 list detail information of the binding sites of p28-azurin, azurin and putative bacteriocins with p53 DBD expressed in Figure 2 and Figure 3. Figure S1 plots the probability score versus odds score for 81 putative bacteriocins expressed in Table S1. Figure S2 presents the models of 14 most putative anticancer azurin-like bacteriocins in cartoon representation. Figure S3 and S4 show the amino acids involved in binding site of docking model of p53 DBD with p28-azurin and azurin respectively.

## Figures and Tables

**Table 1:** Hydrophobic percentages (HPP) of 14 most putative azurin-like bacteriocins.

Number	Sequence ID	BAGEL ID	Bacteria species/strain	Amino acid sequence	HPP (%)
	Azurin		*Pseudomonas aeruginosa* PAO1	MLRKLAAVSLLSLLSAPLLAAECSVDIQGNDQMQFNTNAITVDKSCKQFTVNLSHPGNLPKNVMGHNWVLSTAADMQGVVTDGMASGLDKDYLKPDDSRVIAHTKLIGSGEKDSVTFDVSKLKEGEQYMFFCTFPGHSALMKGTLTLK	49
	p28-azurin			LSTAADMQGVVTDGMASGLDKDYLKPDD	46

1	p2seq05	AOI 1 orf003	*Anaerotruncus colihominis* DSM 17241	MEKPAVESIVKTAGNGTKRWYNRMKKQTAAITAVVCVFLFATHTQALEVPARVTRTVTTTQKIPFATSYIDLPGIYRGYEEPVRSGTPGEQKIEAQVIYEGDRAVKVVSIKAEQTAQPVNAVVKRGTKVLYSETADGSAWKTSFARPLKGGWLSADFYDYPHHNGIDLAAPYGTPVYAAAEGVVEQAGWYGEYGICVILRHADGSRTLYGHNSSVSVSVGQTVKQGEKIANVGSTGNSTGNHLHFEIRVDGRMIDPLVYLDQ	46

2	p3seq17	AOI 1 smallORF 28	*Bacteroides vulgatus* PC510	MFHHSCLVMNIHIWECMSTTLAPQQQRVTLTVITGIVCFLSHTYQTTIRVLALSGTDSFTNNGTTGISA	49
3	p3seq16	AOI 1 smallORF 29		MSTTLAPQQQRVTLTVITGIVCFLSHTYQTTIRVLALSGTDSFTNNGTTGISA	45

4	p3seq24	AOI 1 orf007	*Clostridium hathewayi* DSM 13479	LEELIVNLVQSQGIWAVLFVFLLLYTIKKNDKLDELQEARERKYQELLTQLTVKLSIVNTVNEKLDTIQAVLKEKSD	45

5	p2seq14	AOI 1 smallORF 20	*Clostridium nexile* DSM 1787	MGLLEILPIIPYLLVRACDCTASDRTNTTYSCRRT	46
6	p2seq08	AOI 1 orf006		VERTFSLSKRCYGMSCITTKLEETQLTSIALSVFVTNLFRIQRRILCALLHLFRFWYDRNRYKSWKLQIAA	48
7	p2seq33	AOI 1 orf021		MLKKKVKKYLLISGISFAIGTLGIIFVSVLIEEVVRAIAGEEANKQITQSDLEGLPAWITVEMVQAAIDMMNETGYPASVVLGQMILEAGADGSELANPPYYNCLGQKAHCYKENGTVVMRTEEAWGTVTAEFSTFANYVDCMLAWGNKFTRQPYVDNVTACKRDPVTGHYDADSFITALWKSGYATDPAYVSKVIAVMKSRNLYRFNYMTSADLENGLGEIGTGMFTHPCPGMTYQSSYFGEIREFETGGHKGNDYAAPAGTPTLAAADGTVTVAGWSDSAGNWVVIDHGNGLTTKYMHHSRLLVKTGDTVKKGQQIGEVGSTGQSTGNHLHFQVEENGVPVNPDKYLKGEGNERE	48
8	p2seq32	AOI 1 orf024		MLRKKVKKYLLISGISFAIGILGIIFVSTLIEEVVRAIAGEEANKQIVQSDLDGLPAWITVEMVQAAIDMMNETGYPASVVLGQMILEAGADGSELANPPYYNCLGQKAHCYKESGTVVMRTEEAWGTVTAEFSTFANYVDCMLAWGNKFTRQPYVDNVIACKRDPVTGHYDADSFITALWKSGYATDPAYVSKVIAVMKSRNLYRFNYMTSADLENGLGEIGNGMFTHPCPGMTYQSSYFGEIREFETGGHKGNDYAAPAGTPTLAAADGIVTIAGWSDSAGNWVVIDHGNGLTTKYMHHSKLLVKTGDTVKKGQQIGEVGSTGQSTGNHLHFQVEENGVPVNPDKYLKGDGE	49
9	p2seq18	AOI 1 orf061		VFTSFSLSSIATLPRLKEIILTSCFKHHFKEYTTLSISASPFLSNICILIISLSAKYFFIIAAHIPPCIVVQLSRQKSKLFIMN	51
10	p2seq20	AOI 2 smallORF 1		MVAFVVISPITRTRPVLIATSHATRLSGSCLIHSSNTASAI	51

11	p1seq09	AOI 1 smallORF 1	*Dorea longicatena* DSM 13814	MKRAATCHSHQHLTGLAERYAARHTSGSLQFLFFF	49

12	p1seq16	AOI 1 orf012	*Eubacterium ventriosum* ATCC 27560	NKKSHKSAFVCIAVFMVTMIMILTFYQLAVEIYAYGFIAKNNDYRVKESTAKVLTLKENHKAINIENINVNSKTKVINKKNINVNNFVLKKPVKGGITTSGFGDTISRTASHNGHDWAVNTGTKVRAAAEGVVELAYFSESYGYNILINHNNGFKTRYAHLSEVKVSKGEKVEQSQVIALSGSTGFSTGPHLHFEVVKDGKRVNPIEYVSNR	44

13	p3seq02	AOI 1 smallORF 15	*Ruminococcus* sp. 5.1.39.B FAA	MQVYSLFIFSASVYCKIITGKEDIRCSGTYN	48
14	p3seq04	AOI 1 smallORF 20		MLNAAGCFRISFPTSDSDSLYSSFTVKTVAE	45

**Table 2:** Binding interface properties of 14 bacteriocin models in complex with p53 DBD and comparison with p28-azurin and azurin. The models that have similar binding site with p28-azurin and azurin are shown in bold font.

Number	Sequence ID	Size^(a)^	Same amino acids in binding interface (compared with p28-azurin)^(b)^		Same amino acids in binding interface (compared with azurin)^(c)^		Percentage of polar and nonpolar atoms present in the binding interface of bacteriocin (%)		Percentage of polar and nonpolar atoms present in the binding interface of p53 DBD (%)	
			(number)	(%)	(number)	(%)	Polar	Nonpolar	Polar	Nonpolar
**1**	**p1seq09**	**35**	**16**	**53.33**	**7**	**28.00**	**50.00**	**50.00**	**60.00**	**40.00**
**2**	**p1seq16**	**212**	**16**	**53.33**	**20**	**80.00**	**60.70**	**39.30**	**62.75**	**37.25**
**3**	**p2seq05**	**262**	**17**	**56.67**	**22**	**88.00**	**52.24**	**47.76**	**55.00**	**45.00**
**4**	**p2seq08**	**71**	**10**	**33.33**	**16**	**64.00**	**52.17**	**47.83**	**61.29**	**38.71**
5	p2seq14	35	0	0.00	0	0.00	52.38	47.62	64.29	35.71
6	p2seq18	84	0	0.00	0	0.00	55.56	44.44	63.64	36.36
**7**	**p2seq20**	**41**	**8**	**26.67**	**20**	**80.00**	**50.00**	**50.00**	**64.52**	**35.48**
8	p2seq32	354	0	0.00	0	0.00	57.50	42.50	51.85	48.15
9	p2seq33	357	0	0.00	4	16.00	58.33	41.67	55.17	44.83
**10**	**p3seq02**	**31**	**16**	**53.33**	**8**	**32.00**	**47.37**	**52.63**	**57.69**	**42.31**
11	p3seq04	31	0	0.00	0	0.00	55.56	44.44	64.29	35.71
12	p3seq16	53	0	0.00	0	0.00	45.00	55.00	54.55	45.45
**13**	**p3seq17**	**69**	**11**	**36.67**	**23**	**92.00**	**54.17**	**45.83**	**59.38**	**40.62**
**14**	**p3seq24**	**77**	**14**	**46.67**	**7**	**28.00**	**42.86**	**57.14**	**59.09**	**40.91**

(a) The number of amino acids

(b) The number of amino acids present in the binding interface of p53 DBD that are found in both complexes: p53 DBD/bacteriocin and p53 DBD/p28-azurin. The percentage indicates the percent of this number over the total number of amino acids in the binding interface of p53 DBD/p28-azurin.

(c) The number of amino acids present in the binding interface of p53 DBD that are found in both complexes: p53 DBD/bacteriocin and p53 DBD/azurin. The percentage indicates the percent of this number over the total number of amino acids in the binding interface of p53 DBD/azurin.
