# Supplementary material for: Discovery of Azurin-Like Anticancer Bacteriocins from Human Gut Microbiome through Homology Modeling and Molecular Docking against the Tumor Suppressor p53
Source: Biomed Res Int. 2016 Apr 30;2016:8490482. doi: 10.1155/2016/8490482 (PMC4867070; doi:10.1155/2016/8490482)
Supplement: Supplementary file 1 — Table S1 shows detail information of functional prediction of 81 putative bacteriocins by ProtFun server, probability scores and odd scores. Table S2 shows model assessments for the 14 most putative anticancer azurin-like bacteriocins structure predicted by I-TASSER. Table S3 list detail information of the binding sites of p28-azurin, azurin and putative bacteriocins with p53 DBD expressed in Figure 2 and Figure 3. Figure S1 plots the probability score versus odds score for 81 putative bacteriocins expressed in Table S1. Figure S2 presents the models of 14 most putative anticancer azurin-like bacteriocins in cartoon representation. Figure S3 and S4 show the amino acids involved in binding site of docking model of p53 DBD with p28-azurin and azurin respectively. [file 8490482.f1.pdf]

Supplementary Material

Table S1. Functional prediction of 81 putative bacteriocins by ProtFun server using predicted functions of azurin as reference. The sequences are sorted by odd score from high to low. The higher of the score, the higher of the odds that the sequence belongs to the same categories with the azurin.

No.	Sequence ID	Cellular envelope Probability (a)	Non enzyme Probability (b)	Immune response Probability (c)	Probability product of (a), (b), and (c)	**Log10 (Probability product)**	Cellular envelope Odds	Non enzyme Odds	Immune response Odds	Odds product	**Log10 (Odds product)**
	azurin	0.592	0.818	0.5	2.42E-01	**-0.6**	9.71	1.147	5.877	65.5	**1.8**
1	p2seq07	0.621	0.441	0.345	9.45E-02	**-1.0**	**10.2**	0.6	4.1	**25.5**	**1.4**
2	p1seq19	0.514	0.547	0.189	5.31E-02	**-1.3**	8.4	0.8	2.2	14.4	**1.2**
3	p2seq31	0.237	0.651	0.305	4.71E-02	**-1.3**	3.9	0.9	3.6	12.7	**1.1**
4	p1seq16	0.413	0.515	0.186	3.96E-02	**-1.4**	6.8	0.7	2.2	10.7	**1.0**
5	p1seq02	0.427	0.371	0.18	2.85E-02	**-1.5**	7.0	0.5	2.1	7.7	**0.9**
6	p2seq33	0.416	0.494	0.138	2.84E-02	**-1.5**	6.8	0.7	1.6	7.7	**0.9**
7	p3seq02	0.108	0.486	0.421	2.21E-02	**-1.7**	1.8	0.7	**5.0**	6.0	**0.8**
8	p3seq17	0.1	0.537	0.376	2.02E-02	**-1.7**	1.6	0.8	4.4	5.5	**0.7**
9	p2seq12	0.052	0.778	0.309	1.25E-02	**-1.9**	0.9	1.1	3.6	3.4	**0.5**
10	p3seq29	0.284	0.43	0.091	1.11E-02	**-2.0**	4.7	0.6	1.1	3.0	**0.5**
11	p3seq13	0.041	0.811	0.292	9.71E-03	**-2.0**	0.7	1.1	3.4	2.6	**0.4**
12	p2seq14	0.051	0.721	0.256	9.41E-03	**-2.0**	0.8	1.0	3.0	2.6	**0.4**
13	p2seq24	0.035	0.708	0.363	9.00E-03	**-2.0**	0.6	1.0	4.3	2.4	**0.4**
14	p2seq08	0.175	0.713	0.07	8.73E-03	**-2.1**	2.9	1.0	0.8	2.4	**0.4**

15	p2seq20	0.033	0.8	0.328	8.66E-03	**-2.1**	0.5	1.1	3.9	2.3	**0.4**
16	p3seq09	0.032	0.715	0.369	8.44E-03	**-2.1**	0.5	1.0	4.3	2.3	**0.4**
17	p1seq03	0.029	0.651	0.383	7.23E-03	**-2.1**	0.5	0.9	4.5	2.0	**0.3**
18	p1seq09	0.052	0.653	0.215	7.30E-03	**-2.1**	0.9	0.9	2.5	2.0	**0.3**
19	p2seq30	0.206	0.754	0.047	7.30E-03	**-2.1**	3.4	1.1	0.5	2.0	**0.3**
20	p3seq16	0.03	0.646	0.361	7.00E-03	**-2.2**	0.5	0.9	4.2	1.9	**0.3**
21	p2seq18	0.035	0.535	0.303	5.67E-03	**-2.2**	0.6	0.7	3.6	1.5	**0.2**
22	p2seq32	0.347	0.396	0.041	5.63E-03	**-2.2**	5.7	0.6	0.5	1.5	**0.2**
23	p3seq01	0.035	0.585	0.252	5.16E-03	**-2.3**	0.6	0.8	3.0	1.4	**0.1**
24	p2seq05	0.296	0.415	0.039	4.79E-03	**-2.3**	4.9	0.6	0.5	1.3	**0.1**
25	p1seq18	0.032	0.469	0.297	4.46E-03	**-2.4**	0.5	0.7	3.5	1.2	**0.1**
26	p3seq21	0.032	0.639	0.221	4.52E-03	**-2.3**	0.5	0.9	2.6	1.2	**0.1**
27	p3seq24	0.126	0.788	0.045	4.47E-03	**-2.3**	2.1	1.1	0.5	1.2	**0.1**
28	p3seq04	0.031	0.529	0.263	4.31E-03	**-2.4**	0.5	0.7	3.1	1.2	**0.1**
29	p1seq11	0.029	0.807	0.167	3.91E-03	**-2.4**	0.5	1.1	2.0	1.1	**0.0**
30	p2seq16	0.035	0.493	0.22	3.80E-03	**-2.4**	0.6	0.7	2.6	1.0	**0.0**
31	p2seq01	0.032	0.749	0.138	3.31E-03	**-2.5**	0.5	1.1	1.6	0.9	**0.0**
32	p2seq21	0.06	0.531	0.097	3.09E-03	**-2.5**	1.0	0.7	1.1	0.8	**-0.1**
33	p2seq03	0.032	0.534	0.18	3.08E-03	**-2.5**	0.5	0.7	2.1	0.8	**-0.1**
34	p3seq03	0.048	0.715	0.081	2.78E-03	**-2.6**	0.8	1.0	1.0	0.8	**-0.1**
35	p2seq11	0.033	0.742	0.113	2.77E-03	**-2.6**	0.5	1.0	1.3	0.8	**-0.1**
36	p3seq15	0.032	0.456	0.19	2.77E-03	**-2.6**	0.5	0.6	2.2	0.7	**-0.1**
37	p2seq09	0.032	0.641	0.129	2.65E-03	**-2.6**	0.5	0.9	1.5	0.7	**-0.1**

38	p1seq12	0.037	0.605	0.116	2.60E-03	**-2.6**	0.6	0.8	1.4	0.7	**-0.2**
39	p1seq14	0.033	0.807	0.087	2.32E-03	**-2.6**	0.5	1.1	1.0	0.6	**-0.2**
40	p2seq13	0.033	0.725	0.092	2.20E-03	**-2.7**	0.5	1.0	1.1	0.6	**-0.2**
41	p2seq19	0.046	0.752	0.061	2.11E-03	**-2.7**	0.8	1.1	0.7	0.6	**-0.2**
42	p3seq08	0.092	0.429	0.047	1.85E-03	**-2.7**	1.5	0.6	0.6	0.5	**-0.3**
43	p1seq07	0.032	0.75	0.07	1.68E-03	**-2.8**	0.5	1.1	0.8	0.5	**-0.3**
44	p2seq02	0.171	0.781	0.012	1.60E-03	**-2.8**	2.8	1.1	0.1	0.4	**-0.4**
45	p1seq04	0.039	0.822	0.046	1.47E-03	**-2.8**	0.6	**1.2**	0.5	0.4	**-0.4**
46	p3seq22	0.032	0.687	0.061	1.34E-03	**-2.9**	0.5	1.0	0.7	0.4	**-0.4**
47	p3seq14	0.033	0.804	0.049	1.30E-03	**-2.9**	0.5	1.1	0.6	0.4	**-0.5**
48	p3seq10	0.036	0.581	0.057	1.19E-03	**-2.9**	0.6	0.8	0.7	0.3	**-0.5**
49	p3seq06	0.033	0.759	0.046	1.15E-03	**-2.9**	0.5	1.1	0.5	0.3	**-0.5**
50	p3seq18	0.034	0.774	0.04	1.05E-03	**-3.0**	0.6	1.1	0.5	0.3	**-0.5**
51	p3seq05	0.029	0.703	0.048	9.79E-04	**-3.0**	0.5	1.0	0.6	0.3	**-0.6**
52	p1seq13	0.03	0.631	0.043	8.14E-04	**-3.1**	0.5	0.9	0.5	0.2	**-0.7**
53	p2seq06	0.169	0.3	0.015	7.61E-04	**-3.1**	2.8	0.4	0.2	0.2	**-0.7**
54	p3seq20	0.031	0.627	0.037	7.19E-04	**-3.1**	0.5	0.9	0.4	0.2	**-0.7**
55	p3seq26	0.031	0.82	0.027	6.86E-04	**-3.2**	0.5	1.1	0.3	0.2	**-0.7**
56	p3seq19	0.029	0.784	0.029	6.59E-04	**-3.2**	0.5	1.1	0.3	0.2	**-0.7**
57	p2seq04	0.045	0.744	0.02	6.70E-04	**-3.2**	0.7	1.0	0.2	0.2	**-0.7**
58	p2seq26	0.043	0.78	0.019	6.37E-04	**-3.2**	0.7	1.1	0.2	0.2	**-0.8**
59	p2seq25	0.031	0.808	0.024	6.01E-04	**-3.2**	0.5	1.1	0.3	0.2	**-0.8**
60	p1seq08	0.032	0.784	0.024	6.02E-04	**-3.2**	0.5	1.1	0.3	0.2	**-0.8**

61	p3seq23	0.032	0.657	0.026	5.47E-04	**-3.3**	0.5	0.9	0.3	0.1	**-0.8**
62	p2seq10	0.053	0.8	0.012	5.09E-04	**-3.3**	0.9	1.1	0.1	0.1	**-0.9**
63	p3seq07	0.029	0.71	0.022	4.53E-04	**-3.3**	0.5	1.0	0.3	0.1	**-0.9**
64	p2seq28	0.035	0.762	0.017	4.53E-04	**-3.3**	0.6	1.1	0.2	0.1	**-0.9**
65	p3seq25	0.033	0.773	0.017	4.34E-04	**-3.4**	0.5	1.1	0.2	0.1	**-0.9**
66	p2seq17	0.033	0.729	0.017	4.09E-04	**-3.4**	0.5	1.0	0.2	0.1	**-0.9**
67	p3seq12	0.034	0.517	0.023	4.04E-04	**-3.4**	0.6	0.7	0.3	0.1	**-0.9**
68	p2seq29	0.031	0.698	0.019	4.11E-04	**-3.4**	0.5	1.0	0.2	0.1	**-1.0**
69	p2seq23	0.042	0.733	0.011	3.39E-04	**-3.5**	0.7	1.0	0.1	0.1	**-1.1**
70	p3seq11	0.032	0.729	0.013	3.03E-04	**-3.5**	0.5	1.0	0.1	0.1	**-1.1**
71	p1seq10	0.034	0.774	0.011	2.89E-04	**-3.5**	0.6	1.1	0.1	0.1	**-1.1**
72	p1seq01	0.037	0.22	0.035	2.85E-04	**-3.5**	0.6	0.3	0.4	0.1	**-1.1**
73	p2seq27	0.031	0.801	0.011	2.73E-04	**-3.6**	0.5	1.1	0.1	0.1	**-1.1**
74	p1seq06	0.033	0.78	0.01	2.57E-04	**-3.6**	0.5	1.1	0.1	0.1	**-1.1**
75	p2seq22	0.029	0.705	0.012	2.45E-04	**-3.6**	0.5	1.0	0.1	0.1	**-1.2**
76	p2seq15	0.029	0.786	0.011	2.51E-04	**-3.6**	0.5	1.1	0.1	0.1	**-1.2**
77	p1seq17	0.029	0.681	0.012	2.37E-04	**-3.6**	0.5	1.0	0.1	0.1	**-1.2**
78	p3seq27	0.032	0.568	0.012	2.18E-04	**-3.7**	0.5	0.8	0.1	0.1	**-1.2**
79	p1seq05	0.032	0.28	0.024	2.15E-04	**-3.7**	0.5	0.4	0.3	0.1	**-1.2**
80	p1seq15	0.032	0.406	0.014	1.82E-04	**-3.7**	0.5	0.6	0.2	0.0	**-1.3**
81	p3seq28	0.037	0.371	0.01	1.37E-04	**-3.9**	0.6	0.5	0.1	0.0	**-1.4**

Table S2. Model assessments for the 14 most putative anticancer azurin-like bacteriocins

**No.**	**Sequence ID**	**Before refinement**		**After refinement**	
		Favored rotamers	Ramachandran favored	Favored rotamers	Ramachandran favored
1	p2seq05	73.46%	64.62%	96.21%	88.08%
2	p3seq17	67.74%	50.75%	100.00%	88.06%
3	p3seq16	67.39%	47.06%	95.65%	92.16%
4	p3seq24	83.56%	77.33%	98.63%	96.00%
5	p2seq14	75.00%	54.55%	100.00%	90.91%
6	p2seq08	78.79%	69.57%	96.97%	91.30%
7	p2seq33	72.13%	69.86%	96.86%	89.01%
8	p2seq32	72.89%	70.74%	96.83%	91.48%
9	p2seq18	60.76%	50.00%	100.00%	96.15%
10	p2seq20	74.29%	43.59%	94.29%	79.49%
11	p1seq09	85.71%	69.70%	100.00%	100.00%
12	p1seq16	70.33%	64.76%	96.70%	85.24%
13	p3seq02	78.57%	65.52%	96.43%	93.10%
14	p3seq04	66.67%	34.48%	92.59%	79.31%

Table S3. The binding sites of p28-azurin, azurin and putative bacteriocins with p53 DBD expressed in Figure 2 and Figure 3. The amino acids of p53 DBD which are in binding interface with each bacteriocin are shown in grey color. Note: **SS**: secondary structure, **aaID**: amino acid id of p53 DBD, **azu**: azurin, **p28**: p28-azurin peptide, **109**: p1seq09, **116**: p1seq16, **205**: p2seq05, **208**: p2seq08, **214**: p2seq14, **218**: p2seq18, **220**: p2seq20, **232**: p2seq32, **233**: p2seq33, **302**: p3seq02, **316**: p3seq16, **317**: p3seq17, and **324**: p3seq24

SS	aaID	azu	p28	205	317	316	324	214	208	233	232	218	220	109	116	302
	96															
	97															
	98															
	99															
	100															■
	101						■									
	102						■									■
	103															
	104		■				■							■		■
	105															
	106															
	107															
	108															
	109															
S1	110		■	■			■							■	■	■
	111		■	■			■							■	■	■
	112		■	■			■							■	■	■
Loop 1	113	■	■	■			■							■		■
	114	■	■	■	■				■				■	■	■	■
	115	■	■	■	■				■				■	■	■	■
	116	■	■	■	■				■						■	
	117	■		■	■								■		■	
	118				■										■	
	119	■		■	■								■			
	120				■			■		■	■					
	121							■		■	■	■				
	122	■		■	■								■		■	
	123	■		■	■								■		■	
S2	124	■	■	■											■	
	125		■													
	126		■	■			■							■		■
	127															
	128		■				■							■	■	■
	129		■											■		■
	130		■													
S2'	131						■							■	■	■
	132															
	133		■													
	134															
	135															

	136
	137
	138
	139
	140
S3	141
	142
	143
	144
	145
	146
	147
	148
	149
	150
	151
	152
	153
	154
S4	155
	156
	157
	158
	159
	160
	161
	162
	163
Loop 2	164
	165
	166
	167
	168
	169
	170
	171
	172
	173
	174
	175
	176
H1	177
	178
	179
	180
	181
Loop 2	182
	183
	184
	185
	186
	187
	188

	189															
	190															
	191															
	192															
	193															
	194															
S5	195															
	196															
	197															
	198															
	199															
	200															
	201															
	202															
	203															
S6	204															
	205															
	206															
	207															
	208															
	209															
	210															
	211															
	212															
S7	213															
	214															
	215															
	216															
	217															
	218															
	219															
	220															
	221															
	222															
	223															
	224															
	225															
	226															
	227															
	228															
	229															
S8	230															
	231															
	232															
	233															
	234															
	235															
	236															
Loop 3	237															
	238															
	239															
	240															
	241															

	242															
	243															
	244															
	245															
	246															
	247															
	248															
	249															
	250															
	251															
	252															
	253															
	254															
S9	255															
	256															
	257															
	258															
	259															
	260															
	261															
	262															
	263															
	264															
	265															
	266															
	267															
	268															
S10	269															
	270															
	271															
	272															
	273															
	274															
	275															
	276															
	277															
	278															
	279															
	280															
	281															
	282															
H2	283															
	284															
	285															
	286															
	287															
	288															
	289															

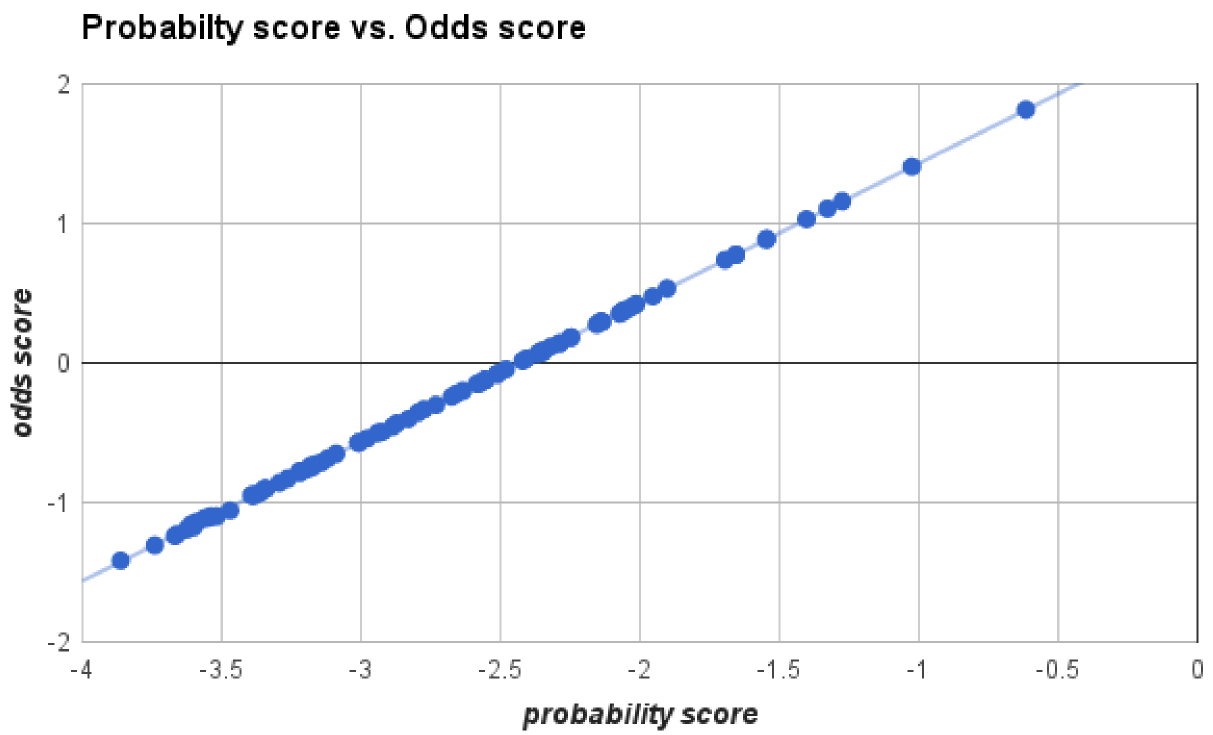

Figure S1. Plot of probability score versus odds score for 81 putative bacteriocins expressed in Table S1

p2seq05<br>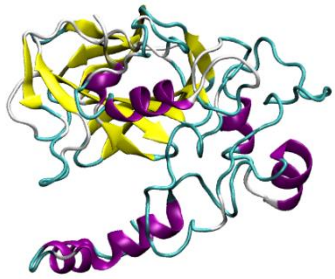	p3seq17<br>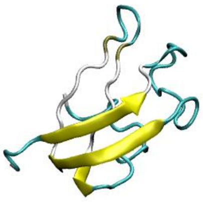
p3seq16<br>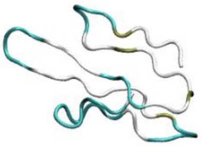	p3seq24<br>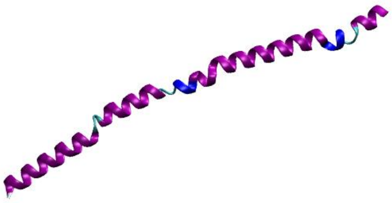
p2seq14<br>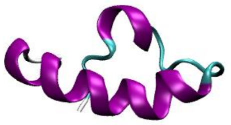	p2seq08<br>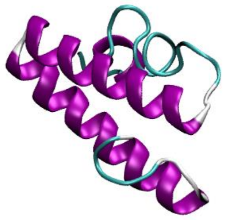
p2seq33	p2seq32

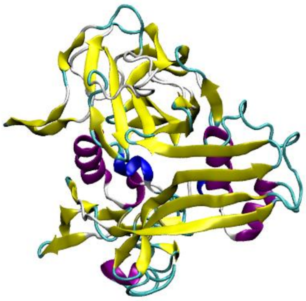	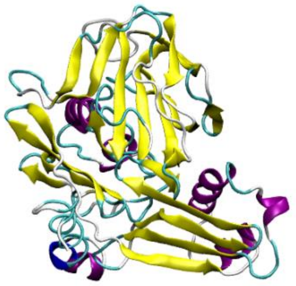
p2seq18	p2seq20
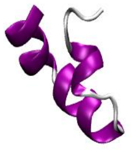	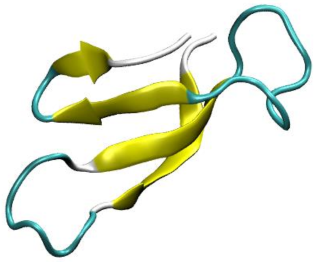
p1seq09	p1seq16
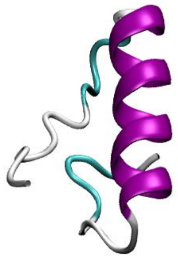	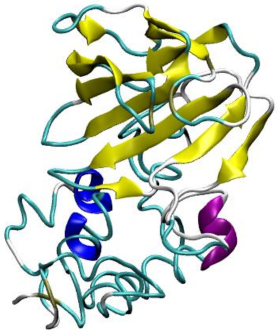
p3seq02	p3seq04

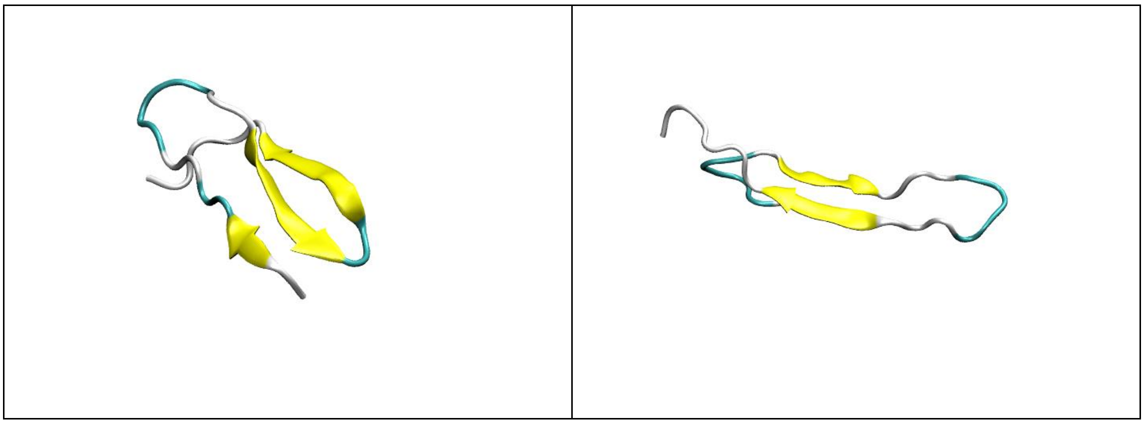

Figure S2. Models of 14 most putative anticancer azurin-like bacteriocins

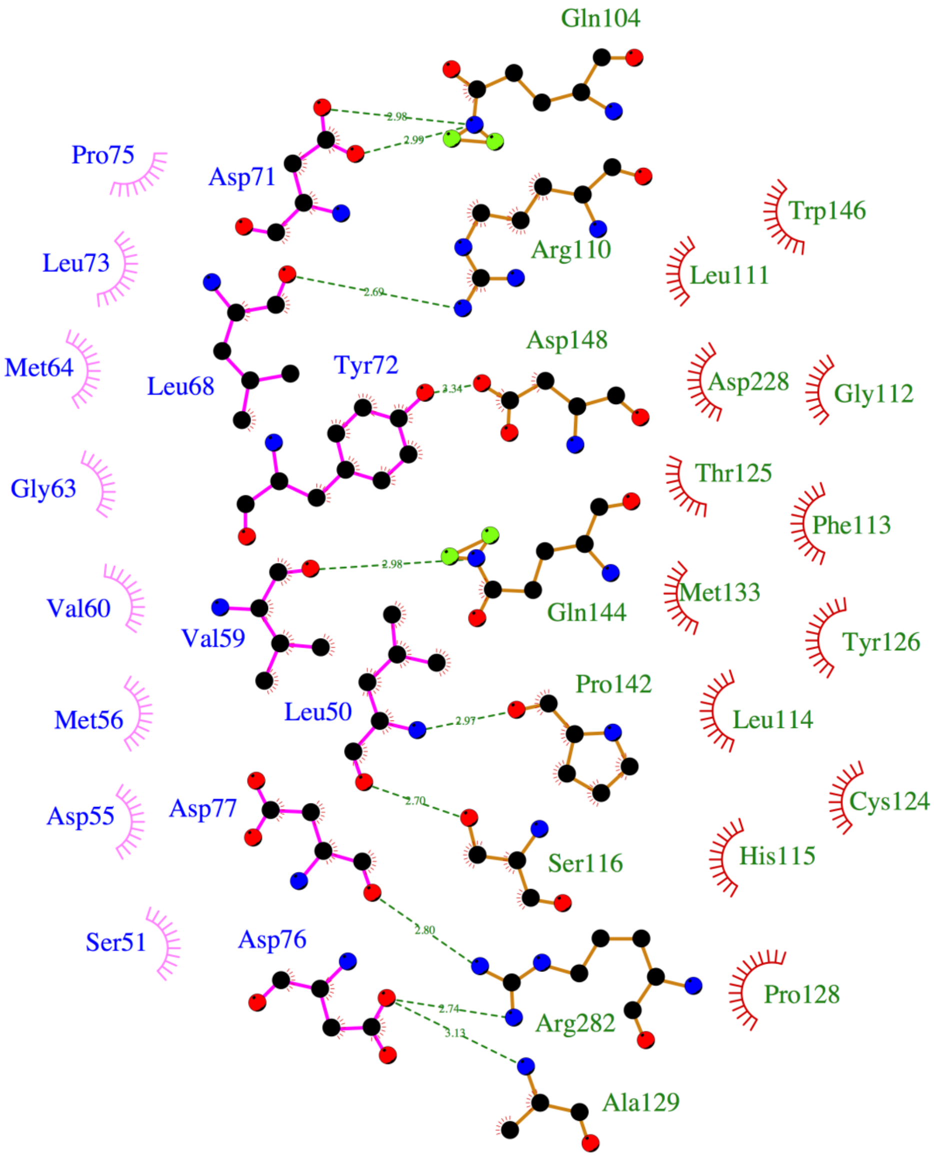

Figure S3. Amino acids involved in binding site of docking model between p53 DBD and p28-azurin. Name of amino acids of p28-azurin are in blue and those of p53 DBD are in green. Hydrogen bonds are shown in dot green line along with molecular details of donors and acceptors.

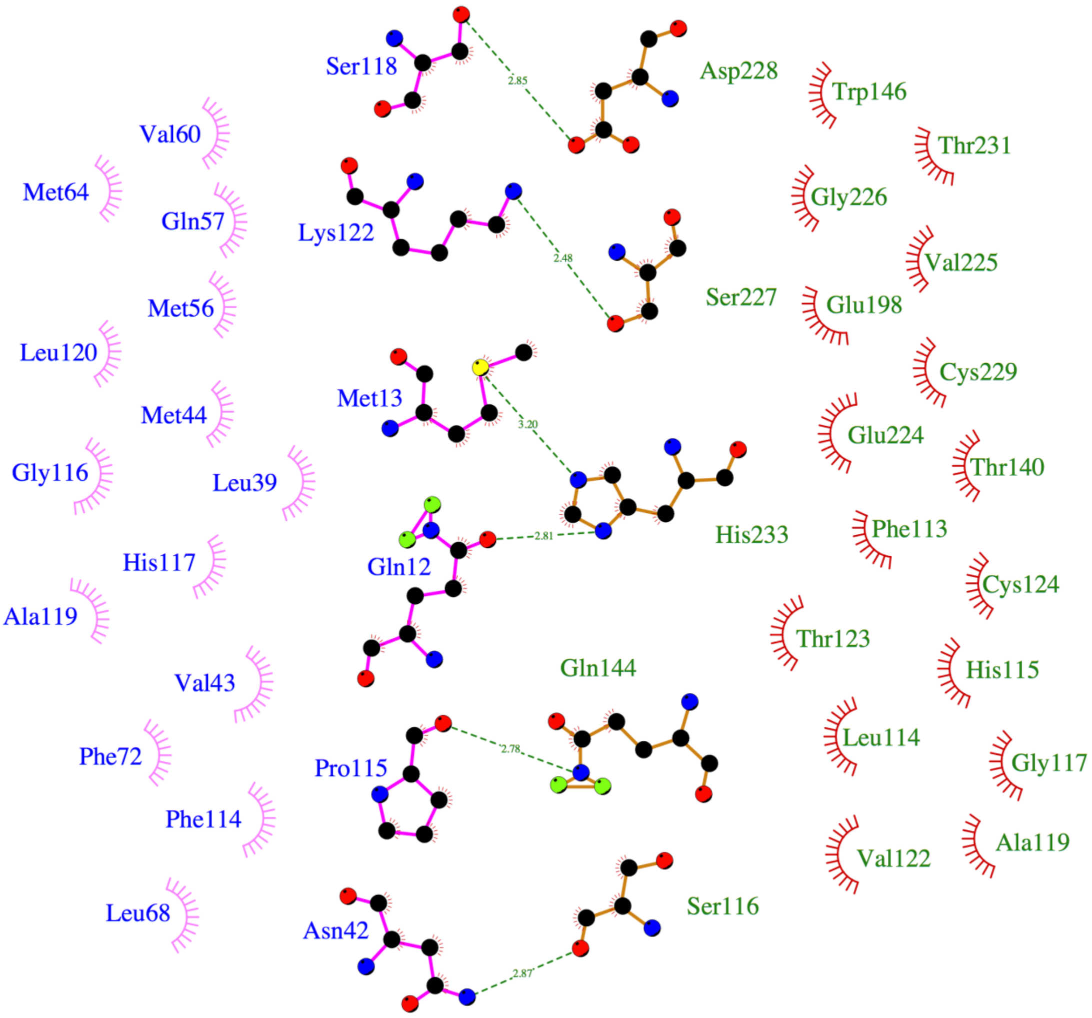

Figure S4. Amino acids involved in binding site of docking model between p53 DBD and azurin. Names of amino acids of azurin are in blue and those of p53 DBD are in green. Hydrogen bonds are shown in dot green line along with molecular details of donors and acceptors.
